# Comparative analysis of two phenotypically-similar but genomically-distinct *Burkholderia cenocepacia*-specific bacteriophages

**DOI:** 10.1186/1471-2164-13-223

**Published:** 2012-06-07

**Authors:** Karlene H Lynch, Paul Stothard, Jonathan J Dennis

**Affiliations:** 1 6-008 Centennial Centre for Interdisciplinary Science, Department of Biological Sciences, University of Alberta, Edmonton, AB, T6G 2E9, Canada; 2 1400 College Plaza, Department of Agricultural, Food and Nutritional Science, University of Alberta, Edmonton, AB, T6G 2C8, Canada

## Abstract

**Background:**

Genomic analysis of bacteriophages infecting the *Burkholderia cepacia* complex (BCC) is an important preliminary step in the development of a phage therapy protocol for these opportunistic pathogens. The objective of this study was to characterize KL1 (vB_BceS_KL1) and AH2 (vB_BceS_AH2), two novel *Burkholderia cenocepacia*-specific siphoviruses isolated from environmental samples.

**Results:**

KL1 and AH2 exhibit several unique phenotypic similarities: they infect the same *B. cenocepacia* strains, they require prolonged incubation at 30°C for the formation of plaques at low titres, and they do not form plaques at similar titres following incubation at 37°C. However, despite these similarities, we have determined using whole-genome pyrosequencing that these phages show minimal relatedness to one another. The KL1 genome is 42,832 base pairs (bp) in length and is most closely related to *Pseudomonas* phage 73 (PA73). In contrast, the AH2 genome is 58,065 bp in length and is most closely related to *Burkholderia* phage BcepNazgul. Using both BLASTP and HHpred analysis, we have identified and analyzed the putative virion morphogenesis, lysis, DNA binding, and MazG proteins of these two phages. Notably, MazG homologs identified in cyanophages have been predicted to facilitate infection of stationary phase cells and may contribute to the unique plaque phenotype of KL1 and AH2.

**Conclusions:**

The nearly indistinguishable phenotypes but distinct genomes of KL1 and AH2 provide further evidence of both vast diversity and convergent evolution in the BCC-specific phage population.

## Background

 The clinical administration of bacteriophages, referred to as phage therapy, has now been used to treat bacterial infections for nearly a century. Although this type of therapy had been largely abandoned outside of Eastern Europe since antibiotics became available in the 1940s, the emergence of antibiotic-resistant pathogens has re-established phage therapy as a viable antibacterial treatment [1]. Recent studies have shown that phages and phage components are effective both in animal models (against species such as *Staphylococcus*, *Pseudomonas*, *Klebsiella*, *Escherichia*, *Salmonella*, and *Campylobacter*) and in human clinical trials [2-8]. Advances in phage delivery and storage (such as nebulization, lyophilization, and spray drying for respiratory phage therapy) and genomic characterization (including high-throughput sequencing and annotation) have made phage therapy more feasible with respect to both logistics and safety [9-12].

One group of bacteria that is thought to be an excellent target for phage therapy is the *Burkholderia cepacia* complex (BCC). These bacterial species, which primarily infect patients with cystic fibrosis (CF), are problematic because they can cause serious illness (including, in up to 20% of cases, a fatal necrotizing pneumonia referred to as ‘cepacia syndrome’), they are capable of patient-to-patient spread (particularly in settings such as CF centers), and, perhaps most importantly, they are highly antibiotic resistant [13-16]. Very few antibiotics are active against the BCC, even in combination: Zhou et al. [16] tested a panel of antibiotics against BCC clinical isolates and determined that less than half of the strains were susceptible to even the most effective drugs. Clinically, the most commonly isolated BCC species are *Burkholderia multivorans* and *Burkholderia cenocepacia*, with the latter thought to be the most pathogenic [17]. BCC phage therapy trials have focused on this species and, to date, phages have been shown to be effective against *B. cenocepacia* in both invertebrate and mammalian infection models [18-20].

As *B. cenocepacia* infections are some of the most problematic for the CF community, the isolation and characterization of novel phages that infect this species remains a priority. Many of these phages have been isolated in recent years, but only some have been fully sequenced (reviewed in [21,22]). Here, we describe the isolation and characterization of KL1 and AH2, two novel *B. cenocepacia*-specific phages with identical host ranges and unique growth characteristics, but strikingly dissimilar genomes.

## Results and discussion

### Isolation, host range and morphology

KL1 was isolated from sewage using *B. cenocepacia* K56-2 as a host. In contrast to enterobacteria phages, which are commonly found in sewage [23], this is the first report of BCC phage isolation from this source. AH2 was isolated from *Nandina* sp. (also known as heavenly bamboo) soil using *B. cenocepacia* C6433. BCC phages have commonly been isolated from both rhizospheres and soil samples, including that of onion and *Dracaena* sp. [18,20,24-27].

**Figure 1:**
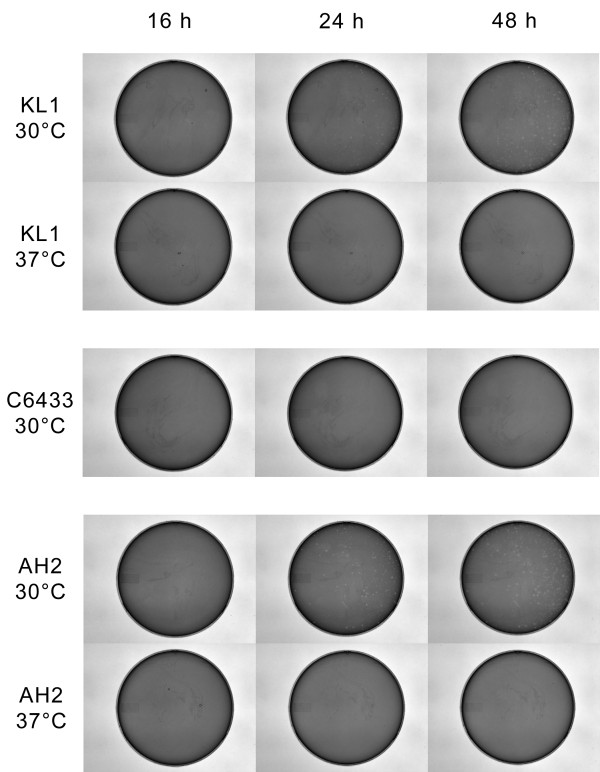
**Development and morphology of KL1 and AH2 plaques.** Phages were plated in half-strength Luria-Bertani (½ LB) agar overlays with a 16 h liquid culture of *Burkholderia cenocepacia* C6433. Plates were incubated at 30°C or 37°C and photographed after 16, 24, and 48 h. C6433 30°C plates (center) are representative of growth at both 30°C and 37°C.

KL1 and AH2 are very similar with respect to both host range and growth characteristics. These phages have a relatively narrow tropism, infecting *B. cenocepacia* K56-2, C6433, 715J, and K63-3. Both KL1 and AH2 exhibit a pattern of lysis that is unique in our collection of BCC-specific phages: although high titre stocks of these phages are very concentrated (up to 10^11^ plaque forming units [PFU]/ml), these phages do not produce clear lysis in agar overlays after 16 h incubation like other phages that we have previously characterized [18,19,26,28-30]. Instead, turbid or no clearing is observed at high titres, with mottling or individual plaques observed at lower titres (approximately 10^7^ PFU/ml or less). At low titres, incubation at 30°C for greater than 16 h is required for plaque formation, but plaques are not observed if incubation is at 37°C (Figure 1). Individual plaques are turbid with a diameter of 0.5-2 mm (larger plaques may have a punctate appearance). When tested with a panel of K56-2 mutants with progressive deficiencies in lipopolysaccharide (LPS) structure (from the O-antigen to the core) [31,32], both phages were able to infect each mutant, suggesting that neither KL1 nor AH2 uses LPS as a major receptor. 

**Figure 2:**
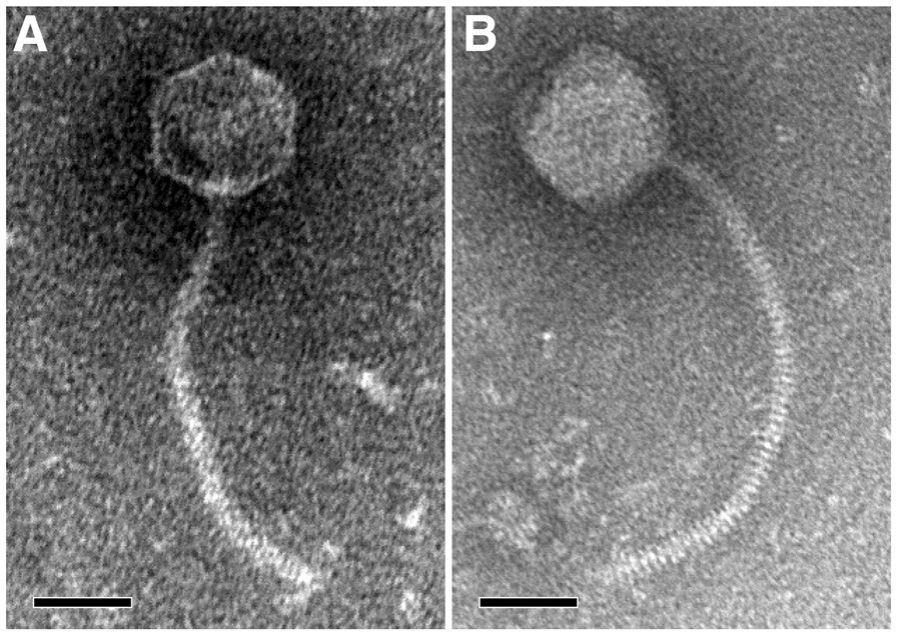
**KL1 (A) and AH2 (B) virion morphology.** Phages were stained with 2% phosphotungstic acid and visualized at 180,000-fold magnification by transmission electron microscopy. Scale bars represent 50 nm.

Both KL1 and AH2 belong to the order *Caudovirales* and family *Siphoviridae* as determined by electron microscopy. The KL1 virion has a non-contractile tail approximately 160 nm in length and a capsid approximately 55 nm in diameter (Figure 2A). The AH2 virion is slightly larger, with a non-contractile tail approximately 220 nm in length and a capsid approximately 60 nm in diameter (Figure 2B). The stacked rings comprising the tail structure are visible in the AH2 micrograph (Figure 2B).

### Genome characterization

**Figure 3:**
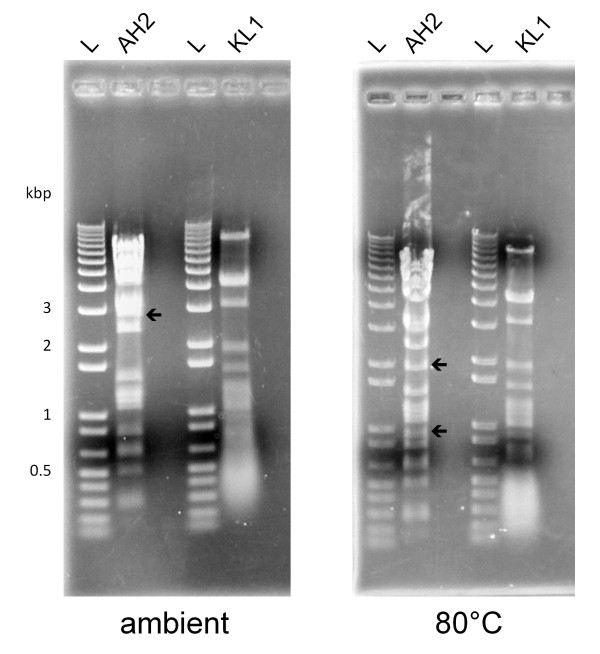
**RFLP analysis of KL1 and AH2 genomic DNA.** 5 μg of genomic DNA were digested overnight with EcoRI and separated on a 0.8% agarose gel. The DNA in the ambient gel (left) was not heated, while the DNA in the 80°C gel (right) was incubated 20 min at 80°C and chilled on ice prior to loading. Arrows indicate bands containing *cos* site DNA. L: 1 Kb Plus DNA Ladder (Invitrogen).

**Figure 4:**
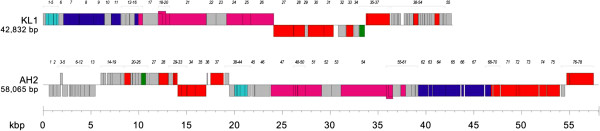
**Circos plots of KL1 and AH2 PROmer comparisons.** Green ribbons indicate regions of similarity between two genomes at the protein level. Each region is on the same strand in both genomes. The scale (in kbp) is shown on the periphery of the plots. PROmer parameters: breaklen = 60, maxgap = 30, mincluster = 20, minmatch = 6. **A**) KL1/AH2 comparison; **B**) KL1/*Pseudomonas* phage 73 (PA73) comparison; **C**) AH2/*Burkholderia* phage BcepNazgul comparison.

Despite the similarities in phenotype between KL1 and AH2 with respect to host range and growth characteristics, the genomes of these two phages are dissimilar. Restriction fragment length polymorphism (RFLP) analysis shows distinct banding patterns of EcoRI-digested KL1 and AH2 genomic DNA, suggesting that their sequences are substantially different (Figure 3). This prediction is confirmed by the results of whole genome pyrosequencing (discussed below) and is illustrated in Figure 4A: in a Circos plot of a PROmer comparison of these two phages, no regions of similarity at the protein level are observed under the parameters used.

**Figure 5:**
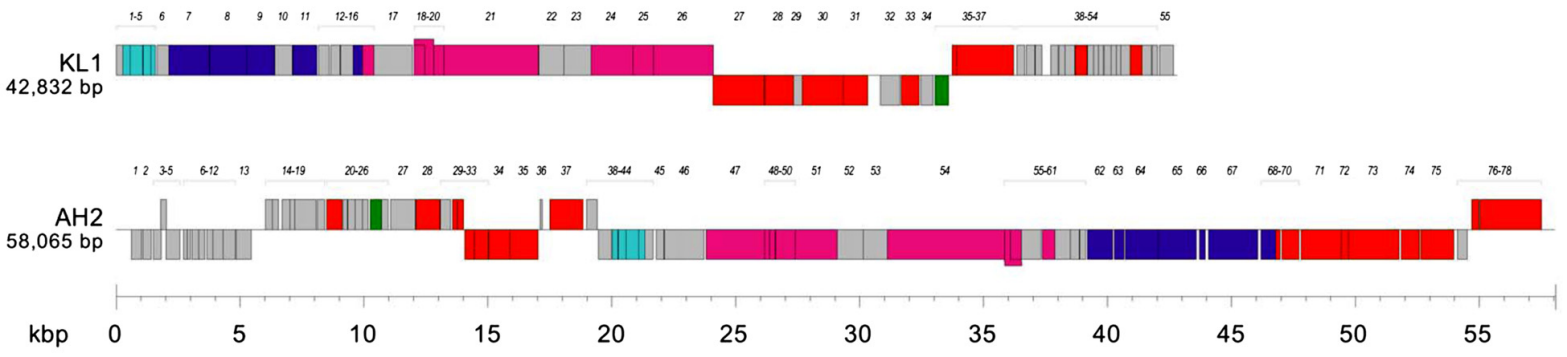
**Genome maps of KL1 and AH2.** Genes transcribed in the forward direction are shown above and those transcribed in the reverse direction are shown below. The scale (in kbp) is shown below the maps. Legend: light blue, lysis; purple, capsid morphogenesis and DNA packaging; pink, tail morphogenesis; red, DNA binding; green, MazG; gray, unknown function.

The KL1 genome is 42,832 base pairs (bp) in length and has a 54.6% GC content. This percentage is lower than that for most *Burkholderia*-specific phages, which tend to have GC contents between 60–65% (excluding phages such as BcepB1A [54.5%], BcepF1 [55.9%], and BcepGomr [56.3%]). We were unable to identify a KL1 *cos* site following incubation of the DNA at 80°C, as the RFLP profiles appeared identical both before and after heating (Figure 3). KL1 is predicted to encode 55 proteins, all of which have an ATG start codon, except for gp2 which has a GTG codon (Figure 5, Table 1).

**Table 1:** KL1 genome annotation

Gene	Start	End	Putative function	Strand	Predicted ribosome binding site and start codon	Length (amino acids)	Closest relative	Alignment region (amino acids)	Percent identity	Source	GenBank accession number
*1*	1	267	unknown	+	AGGGGCGAActtcgtATG	88	hypothetical protein ORF001	1-84/84	77	*Pseudomonas* phage 73	YP_001293408.1
*2*	264	560	holin	+	AAAGGGGCGGtaacGTG	98	hypothetical protein ORF002	3-88/88	42	*Pseudomonas* phage 73	YP_001293409.1
*3*	514	1080	lysin	+	AAAAGGGGttatcgaATG	188	hypothetical protein bglu_1g27070	2-181/188	47	*Burkholderia glumae* BGR1	YP_002912484.1
*4*	1091	1408	Rz	+	AAGTAAGGGGttcgaaATG	105	hypothetical protein ORF004	1-101/101	37	*Pseudomonas* phage 73	YP_001293411.1
*5*	1329	1592	Rz1	+	GAAAGGtgccgccgATG	87	conserved hypothetical protein	1-79/86	40	*Burkholderia* sp. Ch1-1	ZP_06842908.1
*6*	1647	2138	unknown	+	ACTAGGccgcgattATG	163	hypothetical protein ORF005	1-162/162	59	*Pseudomonas* phage 73	YP_001293412.1
*7*	2116	3756	terminase large subunit	+	AACAGGAAttgcttaATG	546	hypothetical protein ORF006	10-531/531	84	*Pseudomonas* phage 73	YP_001293413.1
*8*	3770	5266	portal protein	+	AAAGGAAAcgaaatcATG	498	hypothetical protein ORF007	3-494/501	85	*Pseudomonas* phage 73	YP_001293414.1
*9*	5269	6384	head morphogenesis protein	+	GGGGCGTAatcATG	371	hypothetical protein ORF008	1-364/364	73	*Pseudomonas* phage 73	YP_001293415.1
*10*	6403	7110	unknown	+	AAGGAGtccttgaaATG	235	hypothetical protein ORF009	1-235/239	82	*Pseudomonas* phage 73	YP_001293416.1
*11*	7123	8097	major capsid protein	+	AAGGAcactttatcATG	324	hypothetical protein ORF010	1-325/325	90	*Pseudomonas* phage 73	YP_001293417.1
*12*	8171	8587	unknown	+	AAGGAGtttcgaacATG	138	hypothetical protein ORF011	1-134/134	69	*Pseudomonas* phage 73	YP_001293418.1
*13*	8656	9033	unknown	+	AAAGGAGcgtcgaacATG	125	hypothetical protein ORF012	1-123/123	70	*Pseudomonas* phage 73	YP_001293419.1
*14*	9047	9565	unknown	+	AAGGGGcgcggcatcATG	172	hypothetical protein ORF013	1-172/172	83	*Pseudomonas* phage 73	YP_001293420.1
*15*	9570	9944	head-tail joining protein	+	GATAAGGGtctaacgctATG	124	hypothetical protein ORF014	1-124/126	59	*Pseudomonas* phage 73	YP_001293421.1
*16*	9941	10399	minor tail protein	+	ATACGGTAttgttcgcacaATG	152	hypothetical protein ORF015	5-151/151	68	*Pseudomonas* phage 73	YP_001293422.1
*17*	10412	11965	unknown	+	AAGGAGttacgaaaATG	517	hypothetical protein ORF016	3-511/511	78	*Pseudomonas* phage 73	YP_001293423.1
*18*	12030	12458	tail protein	+	GGAGTAAAccaaATG	142	hypothetical protein ORF017	1-142/142	79	*Pseudomonas* phage 73	YP_001293424.1
*19*	12030	12823	tail protein	+	GGAGTAAAccaaATG	264	hypothetical protein ORF017	1-142/142	79	*Pseudomonas* phage 73	YP_001293424.1
							hypothetical protein ORF018	1-118/118	78	*Pseudomonas* phage 73	YP_001293425.1
*20*	12792	13226	tail protein	+	AAAAGGCGGcgcaacagaATG	144	hypothetical protein ORF019	1-144/144	80	*Pseudomonas* phage 73	YP_001293426.1
*21*	13232	17050	tail tape measure	+	AAGGAttagcagaaATG	1272	hypothetical protein ORF020	1-78, 131-1202/1204	61, 57	*Pseudomonas* phage 73	YP_001293427.1
*22*	17069	18067	unknown	+	AGGAAtacgaattATG	332	hypothetical protein XALc_0225	1-295/307	30	*Xanthomonas albilineans* GPE PC73	YP_003374757.1
*23*	18070	19179	unknown	+	GAGGAAAActaatcATG	369	hypothetical protein ORF033	1-332/333	25	*Pseudomonas* phage M6	YP_001294541.1
*24*	19179	20870	tail assembly protein	+	AAGAAGAtcgcataATG	563	hypothetical protein ORF023	63-565/568	36	*Pseudomonas* phage 73	YP_001293430.1
*25*	20867	21688	tail assembly protein	+	AAGGAcgattccagaATG	273	hypothetical protein ORF024	1-273/274	49	*Pseudomonas* phage 73	YP_001293431.1
*26*	21689	24100	tail assembly protein	+	AAGATGGGGtcggttaaATG	803	hypothetical protein ORF025	1-755/813	49	*Pseudomonas* phage 73	YP_001293432.1
*27*	24097	26166	DNA polymerase	-	AAGGAAtttgcccgATG	689	hypothetical protein ORF026	1-682/683	83	*Pseudomonas* phage 73	YP_001293433.1
*28*	26179	27339	DNA polymerase III β subunit	-	AAGGGGttaaaaATG	386	hypothetical protein ORF027	2-380/380	74	*Pseudomonas* phage 73	YP_001293434.1
*29*	27323	27691	unknown	-	GAATGGtgaaattATG	122	hypothetical protein Dole_2913	5-84/87	33	*Desulfococcus oleovorans* Hxd3	YP_001530793.1
*30*	27696	29351	superfamily II helicase/restriction enzyme	-	AAGGGttacgaATG	551	hypothetical protein ORF029	1-551/551	90	*Pseudomonas* phage 73	YP_001293436.1
*31*	29344	30342	exonuclease	-	GGAAGGcgaagaacgATG	332	hypothetical protein ORF030	1-365/365	65	*Pseudomonas* phage 73	YP_001293437.1
*32*	30852	31637	unknown	-	GAAAGGtgaaacgaacATG	261	hypothetical protein Isop_2441	1-118/151	37	*Isosphaera pallida* ATCC 43644	YP_004179564.1
*33*	31696	32412	recombinase	-	AGGTGAAcgtATG	238	hypothetical protein ORF032	1-238/238	91	*Pseudomonas* phage 73	YP_001293439.1
*34*	32471	32980	unknown	-	AAGGAAccccaaaATG	169	hypothetical protein ORF033	7-146/146	49	*Pseudomonas* phage 73	YP_001293440.1
*35*	33059	33598	pyrophosphohydrolase	-	AGGGGcatcgtATG	179	hypothetical protein ORF034	8-185/185	69	*Pseudomonas* phage 73	YP_001293441.1
*36*	33746	33934	transcriptional regulator	+	GGGGcaagcATG	62	hypothetical protein ORF035	1-61/62	51	*Pseudomonas* phage 73	YP_001293442.1
*37*	33924	36233	primase	+	GAAGGcttgcgcaaatATG	769	hypothetical protein ORF036	1-773/773	85	*Pseudomonas* phage 73	YP_001293443.1
*38*	36366	36668	unknown	+	GAAGGAgttacgaacATG	100	hypothetical protein	132-217/217	44	*Deftia* phage ϕW-14	YP_003359005.1
*39*	36735	37091	unknown	+	GAAGGAGtacacgccATG	118	unnamed protein product	262-336/404	32	*Azospirillum lipoferum* 4B	YP_004974060.1
*40*	37097	37360	unknown	+	AGAAGAAGGAGtaagcgccATG	87	PREDICTED: photosystem II reaction center PSB28 protein, chloroplastic	22-86/179	32	*Vitis vinifera*	XP_002271666.1
*41*	37728	38024	unknown	+	AAAGGAGcgccagccATG	98	hypothetical protein ORF039	1-97/98	70	*Pseudomonas* phage 73	YP_001293446.1
*42*	38060	38296	unknown	+	AAGGAAccccgatcATG	78	hypothetical protein ORF040	1-80/80	50	*Pseudomonas* phage 73	YP_001293447.1
*43*	38302	38703	unknown	+	AAAGGGGtaattactATG	133	hypothetical protein ORF042	1-120/124	40	*Pseudomonas* phage 73	YP_001293449.1
*44*	38707	39195	Vsr endonuclease	+	GACGAAGttgcattaagccATG	162	hypothetical protein ORF043	1-176/179	61	*Pseudomonas* phage 73	YP_001293450.1
*45*	39201	39458	unknown	+	GGAAGGAGtaacccaaATG	85	hypothetical protein Astex_0306	3-81/183	44	*Asticcacaulis excentricus* CB 48	YP_004086155.1
*46*	39455	39655	unknown	+	GGCGAAGtcgtcgaATG	66	monooxygenase, FAD-binding	385-445/546	38	*Streptomyces griseoflavus* Tu4000	ZP_07309792.1
*47*	39652	39840	unknown	+	AAGGAGtacgcaccATG	62	hypothetical protein METUNv1_00516	11-65/68	39	*Methyloversatilis universalis* FAM5	ZP_08503515.1
*48*	39882	40154	unknown	+	AAAAGGAGtaacgaacATG	90	hypothetical protein Cflav_PD2164	58-133/172	30	bacterium Ellin514	ZP_03630603.1
*49*	40138	40374	unknown	+	GAACCGGAttacgattATG	78	hypothetical protein ORF047	2-77/77	67	*Pseudomonas* phage 73	YP_001293454.1
*50*	40374	40550	unknown	+	GGGTTAcgaataATG	58	hypothetical protein Glaag_3667	90-140/227	29	*Glaciecola* sp. 4 H-3-7 + YE-5	YP_004435864.1
*51*	40562	40933	unknown	+	GAAAGGtgaaatcATG	123	hypothetical protein BURMUCGD2M_4586	8-67/70	34	*Burkholderia multivorans* CGD2M	ZP_03569237.1
*52*	40930	41415	dCMP deaminase	+	GGAACGtccggcATG	161	hypothetical protein ORF049	2-153/155	75	*Pseudomonas* phage 73	YP_001293456.1
*53*	41412	41786	unknown	+	AAAGGctgaatcATG	124	hypothetical protein ORF050	4-125/127	43	*Pseudomonas* phage 73	YP_001293457.1
*54*	41826	42032	unknown	+	GGGGAtgcccacattATG	68	hypothetical protein ORF051	37-94/94	45	*Pseudomonas* phage 73	YP_001293458.1
*55*	42120	42674	unknown	+	AAGGAGttttacaaATG	184	hypothetical protein ORF052	9-190/190	66	*Pseudomonas* phage 73	YP_001293459.1

KL1 is most similar to *Pseudomonas* phage 73 (PA73; NC_007806), a siphovirus that infects *Pseudomonas aeruginosa*[33]. These phages are similar with respect to genome length (42,999 bp for PA73 and 42,832 bp for KL1), GC content (53.6% for PA73 and 54.6% for KL1), and predicted number of proteins (52 for PA73 and 55 for KL1). BLASTN comparison of KL1 and PA73 indicates that these sequences are similar over 69% of the KL1 genome. KL1 encodes a protein most similar to each PA73 protein from ORF001–ORF052 (excluding 12 proteins) (Table 1). Most PA73 proteins show limited similarity to others in the NCBI database and have not been assigned a putative function [33]. Of the 9 PA73 proteins with predicted functions, all but one (peptidyl-tRNA hydrolase [peptide chain release factor]) is similar to a KL1 protein: holin, terminase large subunit, head morphogenesis protein, tail tape measure protein, DNA polymerase, superfamily II helicase/restriction enzyme, helicase (annotated here as recombinase), and dCMP deaminase (KL1 gp2, gp7, gp9, gp21, gp27, gp30, gp33, and gp52, respectively) (Table 1). Of the KL1 proteins most similar to a PA73 protein, the most similar is gp33 (91% identity with ORF032) and the least similar is gp24 (36% identity with ORF023) (Table 1). In a Circos plot of a PROmer comparison of these phages, the majority of the two genomes are similar at the protein level (Figure 4B).

The AH2 genome is 58,065 bp in length and has a 61.3% GC content. Incubation of the DNA at 80°C caused a shift in the RFLP profile (Figure 3), suggesting the presence of a *cos* site. Sequencing of the shifted fragments indicates that AH2 has a 12 bp 5’ overhang *cos* site with a sequence almost identical (1 bp difference) to that of *Burkholderia* phage BcepNazgul (NC_005091). AH2 is predicted to encode 78 proteins (Figure 5, Table 2). The majority of the start codons (70) are ATG, 6 are GTG and 2 are TTG (Table 2).

**Table 2:** AH2 genome annotation

Gene	Start	End	Putative function	Strand	Predicted ribosome binding site and start codon	Length (amino acids)	Closest relative	Alignment region (amino acids)	Percent identity	Source	GenBank accession number
*1*	619	1035	unknown	-	AAGGAAAcgacATG	138	hypothetical protein Nazgul32	12-130/130	29	*Burkholderia* phage BcepNazgul	NP_918966.1
*2*	1073	1423	unknown	-	AGGGGGGAAcggccATG	116	conserved hypothetical protein	1-116/116	72	*Burkholderia multivorans* CGD1	ZP_03586942.1
*3*	1501	1818	unknown	-	GGATTActgaccATG	105	family 2 glycosyl transferase	292-387/387	32	*Haloterrigena turkmenica* DSM 5511	YP_003404522.1
*4*	1809	2024	unknown	+	GAGAAAtagagATG	71	mobilization protein mbeA	190-237/325	37	*Escherichia coli* E128010	EFZ49597.1
*5*	2021	2578	unknown	-	AGGGGttacatcATG	185	hypothetical protein Nazgul06	88-158/330	44	*Burkholderia* phage BcepNazgul	NP_919015.1
*6*	2728	2877	unknown	-	AGGTGcaaaaATG	49	hypothetical protein BoklE_20935	6-38/38	48	*Burkholderia oklahomensis* EO147	ZP_02357945.1
*7*	2874	3002	unknown	-	AGGGGcgatcATG	42	polysaccharide deacetylase	21-60/287	35	*Bacillus mycoides* Rock3-17	ZP_04156726.1
*8*	3071	3325	unknown	-	AAAGAgctATG	84	major facilitator superfamily MFS_1	131-209/467	37	*Burkholderia gladioli* BSR3	YP_004349464.1
*9*	3322	3579	unknown	-	GGAGTAtccgccATG	85	hypothetical protein Plabr_1809	308-361/603	31	*Planctomyces brasiliensis* DSM 5305	YP_004269441.1
*10*	3663	3911	unknown	-	GGGGGTAtgacATG	82	HAD-superfamily hydrolase	70-119/268	38	*Methanosphaerula palustris* E1-9c	YP_002465429.1
*11*	3913	4314	unknown	-	AGGGGGAGtaacggccATG	133	hypothetical protein Nazgul09	1-129/141	59	*Burkholderia* phage BcepNazgul	NP_919018.1
*12*	4320	4805	unknown	-	AGGGGttacatcATG	161	hypothetical protein Nazgul10	1-151/160	74	*Burkholderia* phage BcepNazgul	NP_919019.2
*13*	4846	5454	unknown	-	AAAAAGGGGtttttgacATG	202	194 gene product	101-187/188	43	*Salmonella* phage PVP-SE1	YP_004894001.1
*14*	6021	6302	unknown	+	AAGGAGcaatcATG	93	hypothetical protein Nazgul13	3-93/93	41	*Burkholderia* phage BcepNazgul	NP_919022.1
*15*	6311	6550	unknown	+	AGGCGGtcgtATG	79	hypothetical protein BDB_mp60418	1-67/67	45	blood disease bacterium R229	CCA83252.1
*16*	6707	7015	unknown	+	ACACGAcaccATG	102	hypothetical protein MC7420_4162	43-84/88	45	*Microcoleus chthonoplastes* PCC 7420	ZP_05027813.1
*17*	7012	7218	unknown	+	GAAGGtgccggcATG	68	hypothetical protein Cy51472DRAFT_4929	53-81/152	45	*Cyanothece* sp. ATCC 51472	ZP_08976132.1
*18*	7215	8069	unknown	+	AGGAAAGgaaATG	284	hypothetical protein TK90_2682	5-175/177	45	*Thioalkalivibrio* sp. K90mix	YP_003494636.1
*19*	8123	8407	unknown	+	GAGAAGGcacacacATG	94	GTP-binding protein	150-232/1016	29	*Gemmata* sp. Wa1-1	AAX07516.1
*20*	8499	9128	DNA polymerase III β subunit	+	GAACGGTGAGcttATG	209	hypothetical protein Nazgul21	24-216/237	24	*Burkholderia* phage BcepNazgul	NP_918955.1
*21*	9149	9343	unknown	+	AGGAGAAAGgagATG	64	hypothetical protein R2APBS1DRAFT_0277	9-63/344	31	*Rhodanobacter* sp. 2APBS1	ZP_08951135.1
*22*	9346	9645	unknown	+	GGGGGTAtctgaccATG	99	hypothetical protein PFL_2108	3-63/70	33	*Pseudomonas fluorescens* Pf-5	YP_259216.1
*23*	9642	9938	unknown	+	GGAGGGtcaTTG	98	aspA gene product	38-122/317	32	*Rhodospirillum centenum* SW	YP_002297975.1
*24*	9935	10171	unknown	+	GGGGcttggcgtATG	78	hypothetical protein Nazgul19	18-97/97	39	*Burkholderia* phage BcepNazgul	NP_919028.2
*25*	10256	10711	pyrophosphohydrolase	+	AAGGAAAggacATG	151	hypothetical protein BCAS0549	15-139/140	60	*Burkholderia cenocepacia* J2315	YP_002153936.1
*26*	10720	10977	unknown	+	GAGGccggccATG	85	hypothetical protein AGRO_3677	208-273/300	41	*Agrobacterium* sp. ATCC 31749	ZP_08529674.1
*27*	11082	12074	unknown	+	AGGAGAAatcGTG	330	hypothetical protein	8-95/113	48	*Escherichia* phage vB_EcoM_ECO1230-10	ADE87960.1
*28*	12101	13075	transcriptional regulator	+	AAGGAAccgacATG	324	hypothetical protein Pnap_4317	25-252/342	45	*Polaromonas naphthalenivorans* CJ2	YP_973341.1
*29*	13078	13497	unknown	+	GCTGACGAtctctgaccATG	139	hypothetical protein SCHCODRAFT_69044	549-631/848	33	*Schizophyllum commune* H4-8	XP_003030158.1
*30*	13574	13768	transcriptional regulator	+	AGGGAtttttcATG	64	hypothetical protein APT_2164	9-65/75	53	*Acetobacter pasteurianus* NBRC 101655	GAB28674.1
*31*	13768	14031	transcriptional regulator	+	AAGCGGAGccgtcctgATG	87	hypothetical protein Bcep1808_2468	2-85/86	73	*Burkholderia vietnamiensis* G4	YP_001120302.1
*32*	14064	14450	Vsr endonuclease	-	GGAGGAatgATG	128	DNA mismatch endonuclease Vsr	15-141/141	65	*Methylocella silvestris* BL2	YP_002360880.1
*33*	14450	15025	excinuclease	-	AACAGAGttgcagcGTG	191	Excinuclease ABC C subunit domain protein	3-183/192	58	*Pseudomonas syringae* pv. *lachrymans* str. M301315	EGH83133.1
*34*	15038	15892	restriction endonuclease	-	GGCAAAGGtcgccgcATG	284	conserved hypothetical protein	1-285/285	70	*Ralstonia solanacearum* CMR15	CBJ36134.1
*35*	15889	17031	cytosine methylase	-	AGGGGGttcgcGTG	380	DNA-cytosine methyltransferase	1-385/385	66	*Ralstonia solanacearum* CMR15	CBJ36133.1
*36*	17107	17199	unknown	+	ACGAAGccttgcttaATG	30	resistance-nodulation-cell division acriflavin:proton (H+) antiporter	850-868/1014	68	*Bacillus pumilus* SAFR-032	YP_001486844.1
*37*	17511	18842	integrase	+	GAAGGAGGtcttgtagcactgATG	443	chorismate mutase family protein	1-362/386	62	*Phaeobacter gallaeciensis* BS107	ZP_02147383.1
*38*	18990	19412	unknown	+	AAGGAGGAatcATG	140	hypothetical protein Dda3937_00584	60-163/163	40	*Dickeya dadantii* 3937	YP_003882998.1
*39*	19462	20001	unknown	-	GGAGAttttcATG	179	hypothetical protein PcarcW_20243	68-197/198	67	*Pectobacterium carotovorum* subsp. carotovorum WPP14	ZP_03833564.1
*40*	20034	20264	Rz1	-	GGAGGAcgccATG	76	hypothetical protein BURPS668_A2333	27-81/81	62	*Burkholderia pseudomallei* 668	YP_001063327.1
*41*	20277	20588	Rz	-	AGGGGGccgtATG	103	hypothetical protein ORF004	2-101/101	35	*Pseudomonas* phage 73	YP_001293411.1
*42*	20585	21091	lysin	-	AAGGAGAAGAacaGTG	168	hypothetical protein HMPREF0005_02034	1-161/163	60	*Achromobacter xylosoxidans* C54	EFV83908.1
*43*	21088	21339	holin	-	GAAGGGGtggacccgaccATG	83	conserved exported hypothetical protein	1-83/85	35	blood disease bacterium R229	CCA83792.1
*44*	21336	21665	unknown	-	AAGGGGccagaagATG	109	hypothetical protein HDEF_1702	3-87/92	31	Candidatus *Hamiltonella defensa* 5AT (*Acyrthosiphon pisum*)	YP_002924457.1
*45*	21807	22121	unknown	-	AAGGAGAAAtcacATG	104	hypothetical protein PPL19_05085	1-103/161	53	*Pseudomonas psychrotolerans* L19	ZP_09283635.1
*46*	22133	23731	tail fiber protein	-	GGAACGtggacATG	532	hypothetical protein Bpse112_32291	69-240/282	45	*Burkholderia pseudomallei* 112	ZP_02502292.1
*47*	23809	26178	tail assembly protein	-	AGAGGAAGAcaaATG	789	hypothetical protein HCH_05649	2-727/728	34	*Hahella chejuensis* KCTC 2396	YP_436732.1
*48*	26175	26375	tail assembly protein	-	GGGGGCAAgaaATG	66	hypothetical protein HCH_05650	4-67/71	50	*Hahella chejuensis* KCTC 2396	YP_436733.1
*49*	26372	26608	tail assembly protein	-	GAGGActgatcATG	78	putative transmembrane protein	7-82/82	47	*Rhodobacter* sp. SW2	ZP_05845047.1
*50*	26618	27418	tail assembly protein	-	AGGGGGAtcaaacaATG	266	hypothetical protein HCH_05652	1-268/269	39	*Hahella chejuensis* KCTC 2396	YP_436735.1
*51*	27415	29100	tail assembly protein	-	AAGAAGAtcacTTG	561	hypothetical protein HCH_05654	35-560/563	32	*Hahella chejuensis* KCTC 2396	YP_436736.1
*52*	29097	30158	unknown	-	GACGAGGtttgaaATG	353	hypothetical protein D11S_2171	1-326/327	23	*Aggregatibacter actinomycetemcomitans* D11S-1	YP_003256741.1
*53*	30160	31122	unknown	-	GAGCGAGGcataacGTG	320	hypothetical protein XALc_0225	1-194/307	35	*Xanthomonas albilineans* GPE PC73	YP_003374757.1
*54*	31124	35860	tail tape measure	-	GGACTGAAcggaaATG	1578	phage tape measure protein	1-109, 452-1680/1683	33	*Sinorhizobium meliloti* AK83	YP_004548730.1
*55*	35853	36538	tail protein	-	AAGGGGGCGagcATG	228	pre-tape measure frameshift protein G-T	1-242/243	34	*Burkholderia* phage BcepNazgul	NP_918998.2
*56*	36098	36538	tail protein	-	AAGGGGGCGagcATG	146	hypothetical protein Sinme_1368	4-126/142	34	*Sinorhizobium meliloti* AK83	YP_004548729.1
*57*	36549	37337	unknown	-	GAGGAAtcaatcATG	262	hypothetical protein Sinme_1367	1-257/262	45	*Sinorhizobium meliloti* AK83	YP_004548728.1
*58*	37385	37897	minor tail protein	-	GAGGAAAGtataATG	170	hypothetical protein Sinme_1366	7-177/177	50	*Sinorhizobium meliloti* AK83	YP_004548727.1
*59*	37897	38517	unknown	-	GACGCAGGtttgccgacATG	206	hypothetical protein Nazgul55	5-198/205	49	*Burkholderia* phage BcepNazgul	NP_918988.2
*60*	38514	38873	unknown	-	GAGGCGcgtgATG	119	hypothetical protein Sinme_1364	3-120/125	38	*Sinorhizobium meliloti* AK83	YP_004548725.1
*61*	38886	39134	unknown	-	AAAGGAAccatcATG	82	hypothetical protein Nazgul57	1-38/85	47	*Burkholderia* phage BcepNazgul	NP_918990.1
*62*	39205	40233	major capsid protein	-	AAGGAGAAAGcaaaATG	342	capsid protein E	2-343/346	50	*Burkholderia* phage BcepNazgul	NP_918991.1
*63*	40290	40688	decorator protein	-	AGGAGAAccatcATG	132	decorator protein D	4-123/131	49	*Burkholderia* phage BcepNazgul	NP_918992.1
*64*	40743	42071	prohead protease	-	AGGACCAGAAccaATG	442	prohead protease ClpP	4-427/434	53	*Burkholderia* phage BcepNazgul	NP_918994.2
*65*	42068	43591	portal protein	-	GGAAcccgtcgATG	507	phage portal protein	57-554/559	59	*Staphylococcus* phage SA1	ACZ55505.1
*66*	43736	43960	head-tail joining protein	-	GGACAAcactATG	74	head-tail joining protein Lambda W	13-76/76	56	*Burkholderia* phage BcepNazgul	NP_918996.1
*67*	44097	46076	terminase large subunit	-	AAGAcctcgATG	659	terminase large subunit TerL	44-677/677	58	*Burkholderia* phage BcepNazgul	NP_918997.2
*68*	46210	46803	terminase small subunit	-	GAAGGTGAtagcgATG	197	TerS	9-179/222	49	*Burkholderia* phage BcepNazgul	NP_918999.1
*69*	46796	46990	transcriptional regulator	-	AGGAGTAcggtATG	64	aminoglycoside phosphotransferase	423-473/487	29	*Frankia* sp. EUN1f	ZP_06416368.1
*70*	47047	47736	repressor	-	GAAAGGCAAGGcagcagcATG	229	hypothetical protein Rvan_1213	14-180/242	36	*Rhodomicrobium vannielii* ATCC 17100	YP_004011581.1
*71*	47833	49446	helicase	-	ACGAcctcctgcgATG	537	helicase	11-507/522	52	*Burkholderia* phage BcepNazgul	NP_919000.2
*72*	49443	49745	resolvase	-	GAAAGGAGGAttcactGTG	100	conserved phage protein	15-103/108	55	*Burkholderia* phage BcepNazgul	NP_919001.2
*73*	49742	51796	DNA polymerase	-	ACGTcaccATG	684	hypothetical protein ORF026	48-670/683	45	*Pseudomonas* phage 73	YP_001293433.1
*74*	51875	52609	single-stranded DNA binding protein	-	AAAGGTGAcaaaaATG	244	conserved phage protein	4-186/198	35	*Staphylococcus* phage SA1	ACZ55548.1
*75*	52655	53995	Cas4 superfamily exonuclease	-	GATCctctcgaccccATG	446	conserved phage protein	8-448/454	48	*Burkholderia* phage BcepNazgul	NP_919005.2
*76*	54140	54538	unknown	-	GGAGAAatcATG	132	hypothetical protein RUMHYD_01446	1-120/122	26	*Blautia hydrogenotrophica* DSM 10507	ZP_03782010.1
*77*	54718	55017	Cro	+	AACGGAGAtcacaATG	99	hypothetical protein Nazgul73	5-90/97	31	*Burkholderia* phage BcepNazgul	NP_919007.1
*78*	55054	57534	primase	+	GGAGGGgcaATG	826	DR0530-like primase	1-843/843	49	*Burkholderia* phage BcepNazgul	NP_919008.2

AH2 is most similar to BcepNazgul, a siphovirus isolated from soil that infects *Burkholderia ambifaria*. Like PA73 and KL1, these phages are similar with respect to genome length (57,455 bp for BcepNazgul and 58,065 bp for AH2), GC content (60.6% for BcepNazgul and 61.3% for AH2), and predicted number of proteins (73 for BcepNazgul and 78 for AH2). In contrast to KL1 (which is closely related to a single phage), AH2 encodes proteins similar to those from a variety of bacteria and phages (Table 2) and so is less closely related to BcepNazgul than KL1 is to PA73. BLASTN comparison of AH2 and BcepNazgul indicates that these sequences are similar over 16% of the AH2 genome. Twenty-one AH2 proteins are most similar to a BcepNazgul protein (Table 2) and 39 show some similarity based on BLASTP analysis. Of the AH2 proteins most similar to a BcepNazgul protein, the most similar is gp12 (74% identity with Nazgul10) and the least similar is gp20 (24% identity with Nazgul21) (Table 2). In a Circos plot of a PROmer comparison of these phages, the most similar regions at the protein level correspond to AH2 gp12, gp71, gp78 (similar to BcepNazgul Nazgul10, helicase, and DR0530-like primase, respectively) and a portion of the putative capsid morphogenesis and DNA packaging module (Figure 4C).

### Module analysis

#### Overview

We have identified the proteins encoded by KL1 and AH2 as belonging to four different functional categories: virion morphogenesis (including capsid morphogenesis/DNA packaging and tail morphogenesis), lysis, DNA binding (the largest and broadest category), and MazG (a pyrophosphohydrolase [34]). Although the proteins encoded by each phage perform many of the same functions (e.g. both KL1 gp11 and AH2 gp62 are predicted to be major capsid proteins) (Tables 1 and 2), the proteins themselves are dissimilar. As we discuss below, the finding that KL1 and AH2 can create nearly identical phenotypes with two dissimilar sets of proteins may be compelling evidence for convergent evolution occurring in these BCC-specific phages.

#### Virion morphogenesis

Although we have determined that KL1 is a siphovirus (Figure 2A), the identity of many of the structural genes remains unknown. As discussed above, KL1 is most closely related to PA73, a phage whose proteins have largely uncharacterized functions. Based on BLASTP analysis, we have been able to predict the identity of only eight KL1 structural proteins: three involved in capsid morphogenesis and DNA packaging and five involved in tail morphogenesis. Gp7 (terminase large subunit) and gp9 (head morphogenesis protein) are similar to PA73 ORF006 and ORF008, respectively, both of which have been assigned putative functions in the PA73 annotation (Table 1). Gp11 (major capsid protein) is similar to the major capsid proteins of *Escherichia* phage K1H and *Listonella* phage ϕHSIC. Gp20 is similar to tail proteins from multiple *Escherichia* phages including K1G, K1H, and K1ind1-K1ind3. Gp21 is predicted to be the tail tape measure as it is the largest protein encoded by KL1 (1272 amino acids [aa]) and it is similar to the predicted PA73 tape measure protein ORF020 (Table 1). Finally, gp24-gp26 are similar to BcepNazgul tail assembly proteins. Using HHpred analysis, we were able to identify an additional three proteins at a probability threshold of 75%. Gp8 is similar to bacteriophage SPP1 portal protein (99.44% probability), gp15 is similar to λ gpFII head-tail joining protein (82.86% probability), and gp16 is similar to λ gpU minor tail protein (77.70% probability) ( Additional file 1: Table S1).

In comparison with KL1, the structural proteins of AH2 are well defined. Genes *62*–*68* make up the capsid morphogenesis and DNA packaging module, containing genes encoding the major capsid protein, decorator protein, prohead protease, portal protein, head-tail joining protein, and terminase subunits (large and small) (Table 2). Each of these proteins is similar to a BcepNazgul protein, with percent identities between 49-58%. Several genes between *47* and *56* are similar to genes encoding BcepNazgul conserved tail assembly proteins, tape measure protein, and pre-tape measure frameshift protein G-T (with percent identities between 26-38%). Two additional AH2 tail proteins were identified using BLASTP (gp46, similar to *Pseudomonas psychrotolerans* L19 phage tail fiber protein) or HHpred (gp58, similar to λ gpU minor tail protein) analysis ( Additional file 2: Table S2). Hypothetical proteins encoded in this region are likely to be involved in tail morphogenesis based on the proximity of their genes to this module.

**Figure 6:**
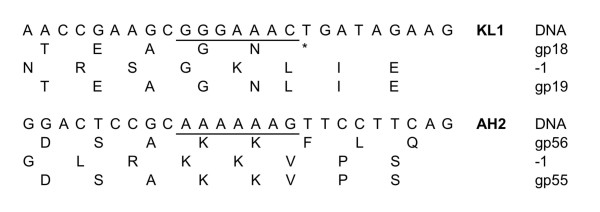
**Sequences of the KL1 and AH2 predicted translational frameshift sites.** For each phage, the first row shows the DNA sequence (with the predicted frameshift site underlined); the second row shows the amino acid sequence in the original frame (the KL1 gp18 stop codon is represented by an asterisk); the third row shows the amino acid sequence in the −1 frame; the fourth row shows the amino acid sequence of the frameshifted protein.

Most tailed phages encode two tail proteins proximal to the tail tape measure gene by way of a −1 translational frameshift [35]. We have previously identified these frameshifted genes in the BCC-specific phages KS9, KS5, KS14, and KL3 [19,29]. Using FSFinder and manual scanning for XXXYYYZ motifs, we predict that KL1 gp18/gp19 and AH2 gp55/gp56 are expressed using this mechanism. The predicted frameshift site in KL1 is GGGAAAC, immediately upstream of the gp18 TGA stop codon (Figure 6 and Additional file 3: Figure S3). A −1 ribosomal shift following the terminal C will allow for expression of the 264 aa gp19 and the 142 aa gp18 from the same start codon (Figure 6). Although most phages encode their frameshifted proteins immediately upstream of the tail tape measure gene, KL1 encodes an intervening tail protein, gp20 (Table 1, Figure 5). This organization is similar to that of *Escherichia coli* phage HK97, *Bacillus subtilis* phage SPP1, *Methanobacterium thermoautotrophicum* phage ψM2, *Methanothermobacter wolfei* phage ψM100, *Lactococcus* phages c2 and BIL67, and *Natrialba magadii* phage ϕch1 [35]. The predicted frameshift site in AH2 is AAAAAAG (Figure 6 and Additional file 3: Figure S3), the same sequence used by *E. coli* phage VT1-Sakai, *M. thermoautotrophicum* phage ψM2, *Staphylococcus aureus* phages PVL and PV83, *Lactococcus lactis* phage ul36, and *Borrelia burgdorferi* prophage Borreliapro [35]. In the case of AH2, a −1 shift of the ribosome following the G in this sequence will allow for the 228 aa gp55 to be expressed instead of the 146 aa gp56 (Figure 6). Using BLASTP or HHpred searches, we were unable to identify the KL1 or AH2 major tail proteins. However, we predict that these proteins may be gp17 in KL1 and gp57 in AH2 as the major tail genes are generally positioned upstream of the frameshifted protein genes [35]. Although not present in all sequences, RNA secondary structures are often found downstream of frameshift sites [19,29,35,36]. Mfold analysis of the 35 bases downstream of the putative KL1 and AH2 sites suggests that stem-loop structures could form in both of these regions ( Additional file 3: Figure S3). 

#### Lysis

In KL1, we have identified the genes putatively encoding the holin, lysin, Rz and Rz1 lysis proteins. In a BLASTP search, gp2 shows similarity to putative holin proteins of PA73 and BcepNazgul. TMHMM analysis of this protein indicates that it has two transmembrane domains, so gp2 is predicted to be a class II holin [37]. Gp3 is similar to the endolysin of *Erwinia* phage vB_EamP-S6 (HQ728266) and contains lysozyme and peptidoglycan-binding conserved domains. Although gp4 does not show similarity to any Rz proteins in the NCBI database, it is predicted to contain a single N-terminal transmembrane domain, characteristic of Rz proteins [38]. Gp5 is predicted to be the KL1 Rz1 protein as it is similar to BcepNazgul Rz1 and LipoP analysis identifies a signal peptidase II cleavage site between positions 17 and 18 (resulting in a 70 aa protein with 4 proline residues [5.7% proline]). The proportion of prolines in the predicted Rz1 lipoprotein is low compared to previously identified Rz1 proteins in BCC phages [19,29,39].

The same lysis proteins were identified in AH2. Like KL1 gp2, the putative AH2 holin gp43 is similar to the BcepNazgul holin, has two transmembrane domains, and is predicted to be a class II holin. Although gp42 shows no similarity to endolysins in a BLASTP search, HHpred analysis reveals similarity to both eukaryotic and prokaryotic lysozyme proteins. Gp41 is predicted to be the AH2 Rz protein as it has a single N-terminal transmembrane domain. Although manual annotation has been required for identification of the Rz1 gene in KL1 and in our previous studies [19,29], we predict that the GeneMark-assigned gp40 is the AH2 Rz1 protein. Gp40 is similar to BcepNazgul Rz1 and has a signal peptidase II cleavage site between amino acids 15 and 16. Similar to the predicted KL1 Rz1, the proportion of prolines present in this protein is relatively low (3/61 or 4.9%). It is unclear from this analysis what protein(s) may contribute to the unique plaque phenotype observed in both of these phages. Aside from the low proportion of proline found in the putative Rz1 proteins, KL1 and AH2 appear to have relatively standard lysis modules, suggesting that unique (and as yet unidentified) proteins may be responsible for controlling lysis timing in each phage.

#### DNA binding

Of the 8 KL1 proteins similar to a PA73 protein with an assigned function, half of these are DNA- or nucleotide-binding proteins: DNA polymerase (gp27), superfamily II helicase/restriction enzyme (gp30), helicase (annotated here as recombinase [gp33]), and dCMP deaminase (gp52) (Table 1). In addition, KL1 encodes a putative DNA polymerase III β subunit (gp28), exonuclease (gp31), transcriptional regulator (gp36), primase (gp37), and Vsr endonuclease (gp44) (Table 1 and Additional file 1: Table S1). In a multi-genome analysis performed by Lopes et al. [40], it was determined that PA73 ORF032 is distantly related to *Lactococcus* phage ϕ31 Sak4 recombinase. When this protein was expressed in *E. coli*, it exhibited recombinase activity, but was found to be less efficient than λ Redβ [40]. Furthermore, PA73 encodes an exonuclease, as is found in characterized phage recombinase pairs such as Redαβ in λ and RecET in *rac*[40]. KL1 gp33 is most closely related to PA73 ORF032 and, with 91% identity, is the KL1 protein most similar to a PA73 protein. In addition, KL1 gp31 has 65% identity with PA73 ORF030 and both of these proteins are similar to λ Redα (99.21% probability for gp31 and 99.17% probability for ORF030) (Table 1 and Additional file 1: Table S1). It is interesting to note that, despite the relatively limited similarity between KL1 and other previously sequenced BCC-specific phages, both gp31 and gp33 are similar to proteins from *Burkholderia* phage BcepGomr (BcepGomrgp43 and BcepGomrgp45, respectively) [40]. Although further characterization of these proteins is required in both KL1 and BcepGomr, it is possible that these exonucleases and Sak4-like recombinases represent a conserved recombination system in certain BCC-specific phages.

AH2 encodes DNA replication, modification, and repair proteins including a putative DNA polymerase III β subunit (gp20), Vsr endonuclease (gp32), excinuclease (gp33), restriction endonuclease/methylase pair (gp34/gp35), integrase (gp37), helicase (gp71), resolvase (gp72), DNA polymerase (gp73), single-stranded DNA binding protein (gp74), Cas4 superfamily exonuclease (gp75), and primase (gp78) (Table 2). Other putative DNA binding proteins are predicted to be involved in transcriptional regulation. Gp28 is similar to partitioning and regulation proteins from *Thermus thermophilus* (100% probability) and *E. coli* (99.86% probability) ( Additional file 2: Table S2). The gp30 and gp31 predicted proteins belong to the helix-turn-helix MerR superfamily and the pyocin activator superfamily, respectively. Both of these proteins, in addition to gp69, also show similarity to excisionase proteins ( Additional file 2: Table S2). Gp70 and gp77 are similar to the lysogeny control proteins CI from enterobacteria phage 186 (99.87% probability) and Cro from *Xylella fastidiosa* Ann-1 (96.60% probability), respectively ( Additional file 2: Table S2).

AH2 gp32-gp35 are predicted to be part of a DNA protection and repair module. Vsr (very short patch repair) endonucleases are involved in the repair of 5-methylcytosine to thymine deamination [41]. Previously, we identified a Vsr endonuclease in the BCC-specific phage KL3 that, along with an EcoRII-C endonuclease/methylase pair, was predicted to be part of a novel non-self DNA degradation and self DNA protection/repair module [29]. Our model proposed that non-KL3 DNA (i.e. that of the host or a superinfecting phage) would be degraded by the endonuclease (gp45), while KL3 DNA would be protected by the methylase (gp47) (converting cytosine to 5-methylcytosine). Vsr endonuclease (gp46) and very short patch repair would then prevent the accumulation of mutations caused by 5-methylcytosine deamination [29].

The DNA protection and repair system of AH2 is analogous to that of KL3. AH2 gp32 has 51% identity with the KL3 Vsr endonuclease and is similar to *E. coli* Vsr endonuclease (100% probability) ( Additional file 2: Table S2). AH2 also encodes an endonuclease/methylase pair: gp34 is similar to *Kluyvera ascorbata* KasI (64% identity) while gp35 is similar to *K. ascorbata* M.KasI, *Brevundimonas diminuta* ATCC 11568 cytosine-specific methyltransferase NlaX, and *Acetobacter pomorum* DM001 modification methylase HpaII (63-66% identity). Gp35 also has several methylase conserved domains, including Dcm (an enzyme that produces 5-methylcytosine bases at sites recognized by Vsr endonuclease) [41]. Gp33 is similar to *Thermotoga maritima* UvrABC system protein C (98.35% probability) and could function together with UvrAB in nucleotide excision repair ( Additional file 2: Table S2) [42]. Although further experiments are required to identify the recognition sites of gp34 and gp35, we predict that this module may function as follows: gp34 cleaves non-self DNA, while self DNA is protected by gp35 methylation and subsequent gp32 repair (with gp33 participating in nucleotide excision repair). Although the identity and arrangement of genes in this module is different in AH2 than in KL3, the identification of a similar module in an unrelated BCC-specific phage suggests that these genes may be widely used for DNA protection and repair in this group of viruses.

#### *MazG*

A notable protein encoded by both KL1 and AH2 is MazG. MazG is a pyrophosphohydrolase that acts on ppGpp, one of the signaling molecules in bacteria produced during the stringent response [43]. When bacterial cells are in an amino acid-limited environment, RelA synthesizes pppGpp, the precursor of ppGpp, and the latter activates the expression of genes required for cell survival (such as *rpoS*) and represses genes required for protein and DNA synthesis (reviewed in [44]). Recently, there has been a great deal of interest in marine phages (especially cyanophages) that encode MazG homologs, such as *Prochlorococcus* phages P-SSM2 and P-SSM4, *Synechococcus* phage S-PM2, *Prochlorococcus* and *Synechococcus* phage Syn9, *Roseobacter* phage SIO1, *Pseudoalteromonas* phage H105/1, almost one-fifth of the cyanophages tested by Bryan et al. [45], and all of the cyanophages analyzed by Sullivan et al. [46][47-51]. It has been suggested that these MazG-encoding phages are better able to infect and propagate within their hosts, which are found in nutrient-limited water. By inactivating ppGpp, these phages can promote the expression of genes that would usually be expressed by an exponential phase cell under nutrient-rich conditions, such as those required for protein and DNA synthesis [52]. There are few published reports of the *mazG* gene in non-marine phages, but it has been previously identified in *Myxococcus* phage Mx8 and mycobacteriophage L5 [45].

The putative MazG proteins encoded by KL1 and AH2 are gp35 and gp25, respectively. KL1 gp35 is similar to putative MazG proteins from phages infecting *Synechococcus* (including S-CRM01, S-SM2, and S-ShM2), *Prochlorococcus* (including P-HM1, P-HM2, and P-SSM2), and *Bacillus* (0305ϕ8-36), as well as to PA73 hypothetical protein ORF034 (Table 1). AH2 gp25 is similar to putative *Clostridium* MazG proteins and to the *Burkholderia* phage proteins ϕE255 gp37, BcepMu gp06, and BcepB1A gp71. Both gp35 and gp25 are similar to *E. coli* MazG (100% and 99.76% probability, respectively) ( Additional file 1: Table S1 and Additional file 2: Table S2). Because BCC bacteria found in soil and water are likely to be nutrient-limited (similar to cyanobacteria), MazG proteins in BCC-specific phages may help to facilitate infection in the environment. This protein may also be involved in the unique plaque phenotype of these phages, as the appearance of plaques at low titre after >16 h incubation (at which time the bacterial lawn appears intact) (Figure 1) suggests that lysis of stationary phase cells may be occurring. Such a trait would be especially important for clinical use, as phage activity may be increased against stationary and/or biofilm cells found in the CF lung.

MazG may also have an effect with respect to BCC pathogenicity. Synthesis of ppGpp has been associated with virulence in species such as *Legionella*, *Listeria*, *Pseudomonas*, *Salmonella*, *Mycobacterium*, and *Vibrio* (although the association in this species has been controversial) [53-59]. In *P. aeruginosa*, *relA* mutants are less virulent than the wildtype when tested in the *Drosophila melanogaster* model [55] and *relA spoT* mutants have reduced antibiotic tolerance [60]. Because MazG activity may mimic the effects of these mutations, it is possible that phage-encoded MazG could modulate the virulence and/or antibiotic tolerance of a lysogen. Further experiments are required to determine if the putative KL1 and AH2 MazG proteins have pyrophosphohydrolase activity, if these genes are expressed in lysogens, and if MazG expression has an effect on pathogenicity.

### Convergent evolution

Although there have been relatively few papers published on the subject, the occurrence of convergent evolution in bacteriophages has been documented previously. Most studies examine the phenomenon at the molecular level by identifying identical base pair and amino acid changes that occur in different phage lineages under the same environmental conditions [61-64]. Structural examples of convergent evolution, such as the *Caudovirales* tail and the tectivirus pseudo-tail, have been reviewed previously [65]. Given the ever-increasing number of completed phage genome sequences, it is expected that many more examples remain to be identified (particularly at the whole genome level). Furthermore, there are likely many examples in the literature of phages with similar phenotypes but dissimilar genomes that have not explicitly been identified as examples of convergent evolution, perhaps because they exhibit what is considered to be a “standard” plaque phenotype.

We predict that KL1 and AH2 represent examples of phage convergent evolution at the whole genome level. As discussed above, these two phages exhibit a plaque phenotype that is both similar and unique in comparison to all other BCC-specific phages that we have characterized previously. Because of these characteristics, KL1 and AH2 were thought to be the same phage prior to RFLP and genomic analysis. However, these phages appear to have convergently evolved because, as discussed throughout, their genomes are almost entirely dissimilar (Figure 4A). The relative rarity of this phenotype among characterized phages of the BCC and other species may be at least partially explained by sampling bias. Standard phage isolation protocols most readily identify those phages that have easily visible plaques on multiple hosts after overnight incubation at a broad range of temperatures. Phages such as KL1 and AH2 may be missed because of poorly visible plaques, incompatible hosts, insufficient incubation times, incorrect temperatures, titres that are too high or too low, overgrowth of bacteria, and/or competition by more rapidly lysing phages. As novel phages continue to be isolated from environmental samples using diverse bacterial hosts, the prevalence, distribution, and genetic basis of this phenotype should become more apparent.

Several mechanisms could explain the delayed plaque formation observed here, including long latent periods or lysis inhibition (both with concomitantly large burst sizes) [66], preferential infection of stationary phase cells, or the gradual release of diffusible lytic enzymes from small plaques. In order to differentiate these possibilities, we performed one-step growth curves for both phages using either exponential or stationary phase C6433 as a host. Using a variation of a standard protocol (described in Methods), the phage titres unexpectedly remained stable (within one order of magnitude) over a 4 h period. Given the uninformative nature of these results, we have thus far been unable to identify the mechanism(s) responsible for the plaque phenotype. Taking into consideration the very specific conditions required for the observation of KL1 or AH2 plaques on solid medium, we predict that the infection kinetics in liquid culture may be highly dependent upon host (both strain and growth phase), incubation time, temperature, titre, and potentially other factors (such as medium) that are not accounted for using standard one-step growth curve protocols.

## Conclusions

A recent publication by Ceyssens et al. [67] provides an interesting counterpoint to our study. While we identified KL1 and AH2 as phages that were phenotypically-similar but genomically-distinct, this group analyzed a set of *Pseudomonas* phages that were phenotypically-distinct but genomically-similar. They found that, among ϕKMV-like viruses with between 83-97% nucleotide identity, there were significant differences observed with respect to latent period, host range, and antibody reactivity [67]. We have made similar observations with our collection of BCC-specific phages: two phages can have distinct phenotypes with respect to liquid clearing and host range while at the same time having almost identical genomes [22]. Taken together, the observations made by Ceyssens et al. [67] and those discussed in this study provide a) novel examples of both divergent and convergent phage evolution and b) further evidence of the broad diversity of phages that infect Gram-negative opportunistic pathogens.

## Methods

### Bacterial strains and growth conditions

*Burkholderia cenocepacia* strains K56-2 and C6433, part of the *Burkholderia cepacia* complex experimental strain panel [68,69], were used for phage isolation and propagation. Strains used for host range analysis (also part of the panel) were acquired from the Belgium Coordinated Collection of Microorganisms LMG Bacteria Collection (Ghent, Belgium) and the Canadian *Burkholderia cepacia* complex Research and Referral Repository (Vancouver, BC). Strains were grown aerobically overnight at 30°C on half-strength Luria-Bertani (½ LB) solid medium or in ½ LB broth with shaking. Lysates for DNA isolation were prepared from soft agar overlays made with ½ LB medium containing agarose instead of agar.

### Phage isolation and propagation

KL1 and AH2 were isolated from sewage and *Nandina* sp. soil, respectively, using standard extraction protocols [26]. Environmental samples were incubated with shaking at 30°C in a slurry of ½ LB broth, suspension medium (SM) (50 mM Tris–HCl [pH 7.5], 100 mM NaCl, 10 mM MgSO_4_, 0.01% gelatin solution), and BCC liquid culture (K56-2 for KL1 isolation and C6433 for AH2 isolation). Solids were pelleted by centrifugation and the supernatant was filter-sterilized, plated in soft agar overlays with the BCC strain used in the extraction, and incubated overnight at 30°C and >24 h at room temperature. Plaques were picked using a sterile Pasteur pipette and transferred into 1 ml SM. Phage propagation was performed using soft agar overlays: 100 μl liquid culture and 100 μl phage stock (diluted in SM if necessary) were incubated 20 min at room temperature, mixed with 3 ml 0.7% ½ LB top agar, overlaid on a plate of ½ LB solid medium, and incubated at 30°C and room temperature until plaque formation was complete. High titre stocks were made by transferring multiple plaques into SM or by overlaying plates with SM and incubating 4–8 h at 4°C on a platform rocker.

### Lysis characterization

Host ranges were performed using soft agar overlays (as described above) or by spotting 10 μl aliquots of phage stock (at multiple dilutions) onto a freshly-plated soft agar overlay containing 100 μl liquid culture. K56-2 LPS mutant [31,32] host ranges were performed similarly using wildtype K56-2, RSF19 (*wbxE*::pRF201), XOA7 (*waaL*::pGP**Ω**Tp), XOA15 (*wabR*::pGP**Ω**Tp), XOA17 (*wabS*::pGPApTp), XOA8 (*wabO*::pGP**Ω**Tp), and CCB1 (*waaC*::pGP**Ω**Tp) (kindly provided by Miguel Valvano).

One-step growth curves were performed using a variation of a standard protocol [39]. One hundred microliters of diluted phage lysate containing 10^6^ PFU of KL1 or AH2 was mixed with 10^8^ colony forming units of C6433 (900 μl 5 h liquid culture [for exponential phase curves] or 100 μl 16 h liquid culture diluted in 800 μl spent ½ LB broth [for stationary phase curves]). The suspension was incubated 15 minutes at 30°C, diluted 1:1000 into a flask containing ½ LB broth (exponential) or spent ½ LB broth (stationary), and incubated without shaking at 30°C. One milliliter samples were withdrawn at one hour intervals for 4 h. Two 100 μl samples were plated immediately in soft agar overlays with C6433. One hundred microliters of chloroform was then added to the sample, mixed 5 s on a vortexer, and separated by centrifugation for 1 min at 13,000 rpm. Two 100 μl chloroform-treated samples were then plated immediately in soft agar overlays with C6433. Plates were incubated 48 h at 30°C prior to plaque enumeration. Experiments were performed in triplicate for each condition (KL1 exponential or stationary phase, AH2 exponential or stationary phase).

### Electron microscopy

Filter-sterilized high titre stocks of KL1 and AH2 were used for electron microscopy. 5–10 μl of phage lysate was deposited onto a carbon-coated copper grid and incubated 5 min at room temperature. Following adsorption of excess lysate onto a filter paper, the grids were stained with 2% phosphotungstic acid for 2 min. Grids were viewed using a Philips/FEI (Morgagni) transmission electron microscope with charge-coupled device camera (University of Alberta Department of Biological Sciences Advanced Microscopy Facility).

### DNA isolation, RFLP analysis, and sequencing

Phage DNA was isolated using polyethylene glycol precipitation and guanidine thiocyanate lysis. One hundred milliliters of phage lysate (propagated on C6433) was collected by overlaying turbid-clear or mottled ½ LB agarose plates with SM and incubating at 4°C 4–8 h on a platform rocker. Following the addition of chloroform, debris in the lysate was pelleted by centrifugation for 10 min at 10,000 rcf and 4°C and the supernatant was filter-sterilized with a Millex-HA 0.45 μm syringe driven filter unit (Millipore, Billerica, MA). Fifty milliliter aliquots of the supernatant were incubated at 37°C ≥40 min with 10 μl DNase I, 10 μl DNase I buffer and 6 μl RNase (Fermentas, Burlington, ON) to degrade contaminating bacterial nucleic acids. Following centrifugation for 10 min at 4000 rcf and 4°C, phages in the supernatant were precipitated in 1 M NaCl and 10% w/v PEG 8000 at 4°C. The precipitated phages were pelleted by centrifugation for 20 min at 10,000 rcf and 4°C and resuspended in 1.6 ml SM. To eliminate residual DNase I activity, the phage suspension was incubated at 37°C 10 min with 40 μl 20 mg/ml proteinase K. Following extraction of the phages with an equal volume of chloroform and the addition of EDTA to 100 mM, ½ volume of 6 M guanidine thiocyanate was added to disrupt the capsids and release the phage DNA. DNA was then purified using the GENECLEAN Turbo Kit (Qbiogene, Irvine, CA). Phage DNA was quantified using a NanoDrop ND-1000 spectrophotometer (Thermo Scientific, Waltham, MA).

RFLP analysis was performed using 5 μg of phage DNA digested overnight at 37°C with EcoRI (Invitrogen, Carlsbad, CA). For *cos* site screening, 5 μg EcoRI digests were incubated 20 min at 80°C, cooled on ice, and separated on 0.8% agarose gels in 1x TAE (pH 8.0). Bands present only in the heated sample were excised from the gel, purified using a GENECLEAN III kit (Qbiogene), cloned into pJET1.2 (Fermentas), and sequenced to identify the *cos* site. Preliminary sequencing of EcoRI phage DNA fragments cloned into pUC19 was performed as described previously [19,29]. For complete genome sequencing, phage DNA was submitted to 454 Life Sciences (Branford, CT) for pyrosequencing. The genome sequences of KL1 and AH2 have been deposited in GenBank with the accession numbers JF939047 and JN564907. Sequence start sites for these files were chosen based on alignment with PA73 for KL1 and at the *cos* site for AH2.

### Bioinformatics analysis

Annotation of the genome sequences and determination of GC contents were performed using GeneMark ( http://exon.biology.gatech.edu/gmhmm2_prok.cgi) [70]. Manual annotations were performed for KL1 *5* (encoding Rz1) and KL1 *19*/AH2 *55* (encoding translationally-frameshifted tail proteins). Homology searches and conserved domain searches were performed using HHpred ( http://toolkit.tuebingen.mpg.de/hhpred) [71] and NCBI’s BLASTN/BLASTP (for full genomes and individual proteins, respectively) ( http://blast.ncbi.nlm.nih.gov) [72] and Conserved Domain Search ( http://www.ncbi.nlm.nih.gov/Structure/cdd/wrpsb.cgi) [73]. FSFinder was used for translational frameshift identification ( http://wilab.inha.ac.kr/fsfinder) [74]. Mfold was used for stem-loop structure identification ( http://mfold.rna.albany.edu/?q=mfold) [75]. Sequence comparisons were visualized using Circos ( http://circos.ca) [76] and PROmer ( http://mummer.sourceforge.net) [77] with the following parameters: breaklen = 60, maxgap = 30, mincluster = 20, minmatch = 6. Lysis protein analysis was performed using TMHMM for transmembrane region identification ( http://www.cbs.dtu.dk/services/TMHMM) [78] and LipoP for signal peptidase II cleavage site identification ( http://www.cbs.dtu.dk/services/LipoP) [79].

## Competing interests

The authors declare that they have no competing interests.

## Authors’ contributions

KHL isolated KL1, performed electron microscopy, sequenced, annotated, and analyzed the genomes, and drafted the manuscript. PS constructed Figure 4 and performed FSFinder and preliminary HHpred analysis. JJD devised the study and assisted with experimental design, data analysis, and the writing of the manuscript. All authors read and approved the final manuscript.

## Supplementary Material

Additional file 1 **Table S1. **KL1 HHpred predictions. Click here for file

Additional file 2 **Table S2.** AH2 HHpred predictions. Click here for file

Additional file 3 **Figure S3.** Stem-loop structures predicted by mfold analysis of the KL1 (left) and AH2 (right) frameshift regions (including the putative frameshift sites and 35 downstream bases). Click here for file
