# Supplementary material for: Comparative analysis of two phenotypically-similar but genomically-distinct Burkholderia cenocepacia-specific bacteriophages
Source: BMC Genomics. 2012 Jun 7;13:223. doi: 10.1186/1471-2164-13-223 (PMC3483164; doi:10.1186/1471-2164-13-223)
Supplement: Additional file 1 — Table S1. KL1 HHpred predictions. [file 1471-2164-13-223-S1.docx]

Table S1: KL1 HHpred predictions

| Protein | Motif of closest relative | Motif definition | Probability (%) | E-value |
| --- | --- | --- | --- | --- |
| gp1 | 3ghg_A | Fibrinogen alpha chain | 84.48 | 4.1 |
| gp2 | 2w9y_A | CE-FAR-7, fatty acid/retinol binding protein protein 7, isoform A | 26.14 | 33 |
| gp3 | 2ikb_A | Hypothetical protein NMB1012 | 100.00 | 0 |
| gp4 | 2o2z_A | Hypothetical protein | 47.27 | 4.3 |
| gp5 | 2w9x_A | AXE2A, CJCE2B, putative acetyl xylan esterase | 54.01 | 2.7 |
| gp6 | 3bkh_A | Phikz144, lytic transglycosylase | 60.54 | 7.3 |
| gp7 | 2wbn_A | G2P, terminase large subunit | 99.93 | 1.5e^-26^ |
| gp8 | 2jes_A | Portal protein | 99.44 | 2.5e^-12^ |
| gp9 | 2hl7_A | Cytochrome C-type biogenesis protein CCMH | 27.60 | 72 |
| gp10 | 3iox_A | AGI/II, PA | 42.97 | 1.1e^+02^ |
| gp11 | 1ohg_A | Major capsid protein | 97.56 | 0.0004 |
| gp12 | 2puz_A | Imidazolonepropionase | 54.92 | 5 |
| gp13 | 1epu_A | S-SEC1 | 20.83 | 90 |
| gp14 | 1xn8_A | Hypothetical protein YQBG | 92.97 | 0.027 |
| gp15 | 1k0h_A | Gpfii | 82.86 | 0.7 |
| gp16 | 2l25_A | Uncharacterized protein | 99.96 | 2.1e^-29^ |
| gp17 | 2h0e_A | Transthyretin-like protein PUCM | 8.76 | 5.9e^+02^ |
| gp18 | 3klu_A | Uncharacterized protein YQBN | 61.50 | 5.5 |
| gp19 | 3klu_A | Uncharacterized protein YQBN | 67.45 | 3.2 |
| gp20 | 1j7g_A | D-tyrosyl-tRNA(Tyr) deacylase | 22.47 | 53 |
| gp21 | 2ch7_A | Methyl-accepting chemotaxis protein | 96.85 | 0.53 |
| gp22 | 1ed7_A | Chitinase A1, (CHBD-CHIA1) | 29.49 | 15 |
| gp23 | 1wh7_A | ZF-HD homeobox family protein | 55.37 | 4.3 |
| gp24 | 2e6j_A | Hydin protein | 81.28 | 4.4 |
| gp25 | 3ejh_E | Collagen type-I A1 chain | 52.78 | 3.8 |
| gp26 | 3d37_A | Tail protein, 43 kDa | 97.01 | 0.011 |
| gp27 | 2gv9_A | DNA polymerase | 100.00 | 0 |
| gp28 | 3d1g_A | DNA polymerase III subunit beta | 98.71 | 1.2e^-09^ |
| gp29 | 2waq_P | DNA-directed RNA polymerase RPO12 subunit | 58.55 | 3 |
| gp30 | 1oyw_A | RECQ helicase, ATP-dependent DNA helicase | 100.00 | 0 |
| gp31 | 1avq_A | RED alpha, lambda exonuclease | 99.21 | 2.9e^-10^ |
| gp32 | 3pgw_B | SM B | 85.48 | 13 |
| gp33 | 2cvh_A | DNA repair and recombination protein RADB | 98.66 | 5.2e^-08^ |
| gp34 | 1ez0_A | ALDH, aldehyde dehydrogenase | 44.15 | 15 |
| gp35 | 3cra_A | Protein MAZG | 100.00 | 0 |
| gp36 | 3kjx_A | Transcriptional regulator, LACI family | 95.68 | 0.0082 |
| gp37 | 3m1m_A | ORF904 | 96.05 | 0.034 |
| gp38 | 3acd_A | Hypoxanthine-guanine phosphoribosyltransferase | 23.48 | 13 |
| gp39 | 2cpr_A | Exosome component 10 | 48.54 | 7.9 |
| gp40 | 3kp1_E | D-ornithine aminomutase S component | 27.50 | 9.1 |
| gp41 | 1ejx_B | Urease beta subunit | 26.38 | 7.3 |
| gp42 | 1yg0_A | COP associated protein | 72.76 | 2.7 |
| gp43 | 1rya_A | GDP-mannose mannosyl hydrolase | 52.18 | 7.2 |
| gp44 | 1vsr_A | Protein (VSR endonuclease) | 78.74 | 14 |
| gp45 | 2cnd_A | NADH-dependent nitrate reductase | 43.63 | 3.4 |
| gp46 | 1kca_A | Repressor protein CI | 47.83 | 16 |
| gp47 | 3lyv_A | Ribosome-associated factor Y | 87.21 | 0.74 |
| gp48 | 1gka_B | Crustacyanin A2 subunit | 54.97 | 4.2 |
| gp49 | 1zso_A | Hypothetical protein | 47.38 | 6.1 |
| gp50 | 2etd_A | LEMA protein | 57.81 | 5.1 |
| gp51 | 3gf8_A | Putative polysaccharide binding proteins (DUF1812) | 33.32 | 10 |
| gp52 | 2hvw_A | Deoxycytidylate deaminase | 100.00 | 7.4e^-44^ |
| gp53 | 2o4d_A | Hypothetical protein PA0269 | 69.12 | 5.3 |
| gp54 | 3lay_A | Zinc resistance-associated protein | 86.95 | 0.24 |
| gp55 | 3db3_A | E3 ubiquitin-protein ligase UHRF1 | 44.24 | 5.3 |
