# Supplementary material for: Comparative analysis of two phenotypically-similar but genomically-distinct Burkholderia cenocepacia-specific bacteriophages
Source: BMC Genomics. 2012 Jun 7;13:223. doi: 10.1186/1471-2164-13-223 (PMC3483164; doi:10.1186/1471-2164-13-223)
Supplement: Additional file 2 — Table S2. AH2 HHpred predictions. [file 1471-2164-13-223-S2.docx]

Table S2: AH2 HHpred predictions

| Protein | Motif of closest relative | Motif definition | Probability (%) | E-value |
| --- | --- | --- | --- | --- |
| gp1 | 2vch_A | Hydroquinone glucosyltransferase | 35.45 | 49 |
| gp2 | 3hma_A | N-acetylmuramoyl-L-alanine amidase XLYA | 40.29 | 21 |
| gp3 | 1cii_A | Colicin IA | 50.72 | 6.1 |
| gp4 | 3lnr_A | Aerotaxis transducer AER2 | 37.48 | 28 |
| gp5 | 1z8u_A | Alpha-hemoglobin stabilizing protein | 17.95 | 49 |
| gp6 | 2ov8_A | STAL | 30.13 | 18 |
| gp7 | 2xwp_A | Sirohydrochlorin cobaltochelatase | 29.29 | 11 |
| gp8 | 3fvv_A | Uncharacterized protein | 43.25 | 4.9 |
| gp9 | 3bus_A | REBM, methyltransferase | 70.81 | 1.7 |
| gp10 | 1p9l_A | Dihydrodipicolinate reductase | 28.60 | 29 |
| gp11 | 2wy4_A | Single domain haemoglobin | 51.19 | 19 |
| gp12 | 3cry_A | Gamma-glutamyl cyclotransferase | 31.17 | 19 |
| gp13 | 2k5e_A | Uncharacterized protein | 60.14 | 5.4 |
| gp14 | 1vqy_A | Hypothetical protein AGR_PAT_315 | 24.40 | 62 |
| gp15 | 2dk5_A | DNA-directed RNA polymerase III 39 kDa polypeptide | 30.05 | 13 |
| gp16 | 2d9z_A | Protein kinase C, NU type | 53.00 | 6 |
| gp17 | 3cl6_A | PUUE allantoinase | 40.51 | 6.2 |
| gp18 | 3eqe_A | Putative cystein deoxygenase | 92.50 | 0.86 |
| gp19 | 1u5p_A | Spectrin alpha chain, brain | 27.41 | 72 |
| gp20 | 1vpk_A | DNA polymerase III, beta subunit | 90.54 | 0.74 |
| gp21 | 2rqp_A | Heterochromatin protein 1-binding protein 3 | 45.74 | 18 |
| gp22 | 1rrz_A | Glycogen synthesis protein GLGS | 60.01 | 6.2 |
| gp23 | 3eyt_A | Uncharacterized protein SPOA0173 | 64.51 | 2.8 |
| gp24 | 1o66_A | 3-methyl-2-oxobutanoate hydroxymethyltransferase | 76.36 | 1.4 |
| gp25 | 3cra_A | Protein MAZG | 99.76 | 1.6e^-18^ |
| gp26 | 3p4h_A | ATP-dependent DNA ligase, N-terminal domain prote | 57.99 | 1 |
| gp27 | 3hd5_A | Thiol:disulfide interchange protein DSBA | 41.73 | 19 |
| gp28 | 1vz0_A | PARB, chromosome partitioning protein PARB | 100.00 | 1.4e^-36^ |
| gp29 | 3i23_A | Oxidoreductase, GFO/IDH/MOCA family | 67.39 | 1.5 |
| gp30 | 1z4h_A | TORI, TOR inhibition protein | 98.09 | 2.5e^-06^ |
| gp31 | 1y6u_A | XIS, excisionase from transposon TN916 | 95.91 | 0.0032 |
| gp32 | 1cw0_A | Protein (DNA mismatch endonuclease) | 100.00 | 0 |
| gp33 | 1yd0_A | Uvrabc system protein C | 98.35 | 3.8e^-07^ |
| gp34 | 3bxp_A | Putative lipase/esterase | 55.34 | 19 |
| gp35 | 3g7u_A | Cytosine-specific methyltransferase | 100.00 | 0 |
| gp36 | 1qnr_A | Endo-1,4-B-D-mannanase | 60.93 | 1.1 |
| gp37 | 1z1b_A | Integrase | 100.00 | 3.6e^-35^ |
| gp38 | 2qjz_A | Microtubule-associated protein RP/EB family member 1 | 83.86 | 0.68 |
| gp39 | 2bt9_A | Lectin | 52.04 | 20 |
| gp40 | 1rlf_A | RLF, RLF-RBD | 35.11 | 41 |
| gp41 | 3bwh_A | Cucurmosin | 31.60 | 24 |
| gp42 | 1gbs_A | Australian black SWAN egg white lysozyme | 90.90 | 0.062 |
| gp43 | 2w9y_A | CE-FAR-7, fatty acid/retinol binding protein protein 7, isoform A | 46.11 | 11 |
| gp44 | 3cve_A | Homer protein homolog 1 | 75.30 | 13 |
| gp45 | 1uii_A | Geminin | 77.96 | 4.1 |
| gp46 | 2yyo_A | SPRY domain-containing protein 3 | 99.85 | 6.7e^-21^ |
| gp47 | 3cdd_A | Prophage MUSO2, 43 kDa tail protein | 97.10 | 0.011 |
| gp48 | 2d5r_A | CCR4-NOT transcription complex subunit 7 | 85.46 | 0.41 |
| gp49 | 2vup_A | Glutathione peroxidase-like protein | 50.86 | 7.9 |
| gp50 | 3d37_A | Tail protein, 43 kDa | 80.25 | 8 |
| gp51 | 2ys4_A | Hydrocephalus-inducing protein homolog | 57.73 | 12 |
| gp52 | 1wh7_A | ZF-HD homeobox family protein | 33.02 | 17 |
| gp53 | 1ed7_A | Chitinase A1, (CHBD-CHIA1) | 28.59 | 16 |
| gp54 | 2ch7_A | Methyl-accepting chemotaxis protein | 97.16 | 0.44 |
| gp55 | 2b8i_A | PAS factor | 31.46 | 16 |
| gp56 | 2b8i_A | PAS factor | 61.55 | 6.1 |
| gp57 | 3hcj_A | MSRB, peptide methionine sulfoxide reductase | 18.36 | 1.2e^+02^ |
| gp58 | 3fz2_A | Minor tail protein U | 88.10 | 0.21 |
| gp59 | 2wq1_A | General control protein GCN4 | 40.60 | 41 |
| gp60 | 2kbn_A | Conserved protein | 53.65 | 21 |
| gp61 | 1cc5_A | Cytochrome C5 | 59.68 | 2.9 |
| gp62 | 3bqw_A | Putative capsid protein of prophage | 100.00 | 0 |
| gp63 | 1td4_A | Head decoration protein | 99.94 | 1.3e^-27^ |
| gp64 | 3bf0_A | Protease 4 | 100.00 | 0 |
| gp65 | 2jes_A | Portal protein | 98.33 | 4.4e^-05^ |
| gp66 | 1hyw_A | GPW, head-TO-tail joining protein W | 99.57 | 6.2e^-15^ |
| gp67 | 2o0j_A | Terminase, DNA packaging protein GP17 | 98.70 | 5.7e^-08^ |
| gp68 | 1j9i_A | GPNU1 DBD | 97.58 | 0.00012 |
| gp69 | 1z4h_A | TORI, TOR inhibition protein | 84.08 | 0.33 |
| gp70 | 2fjr_A | Repressor protein CI | 99.87 | 7.5e^-22^ |
| gp71 | 1z3i_X | Similar to RAD54-like | 100.00 | 0 |
| gp72 | 2wcw_A | HJC | 97.87 | 0.00011 |
| gp73 | 3iay_A | DNA polymerase delta catalytic subunit | 100.00 | 0 |
| gp74 | 1je5_A | Helix-destabilizing protein | 99.83 | 2.9e^-20^ |
| gp75 | 3l0a_A | Putative exonuclease | 99.79 | 4e^-19^ |
| gp76 | 2ghj_A | 50S ribosomal protein L20 | 25.67 | 52 |
| gp77 | 3bd1_A | CRO protein | 96.60 | 0.0015 |
| gp78 | 1ro2_A | Hypothetical protein ORF904 | 99.83 | 6.2e^-21^ |
