# Supplementary figures and images for: Comparative analysis of two phenotypically-similar but genomically-distinct Burkholderia cenocepacia-specific bacteriophages
Source: BMC Genomics. 2012 Jun 7;13:223. doi: 10.1186/1471-2164-13-223 (PMC3483164; doi:10.1186/1471-2164-13-223)

## Slide 1
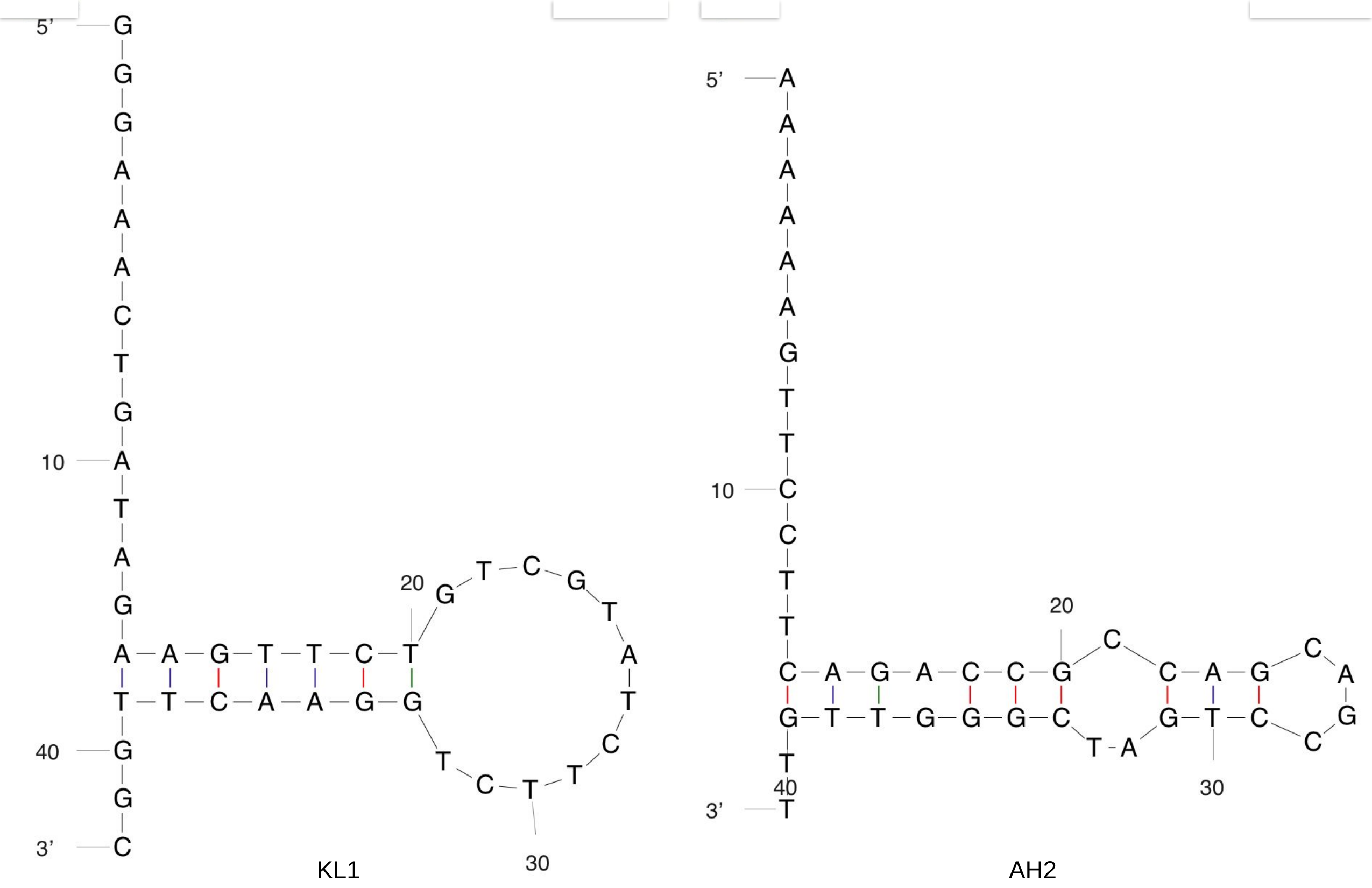

KL1
AH2

Supplement: Additional file 3 — Figure S3. Stem-loop structures predicted by mfold analysis of the KL1 (left) and AH2 (right) frameshift regions (including the putative frameshift sites and 35 downstream bases). [file 1471-2164-13-223-S3.pptx]
